# Overview of HCV Life Cycle with a Special Focus on Current and Possible Future Antiviral Targets

**DOI:** 10.3390/v11010030

**Published:** 2019-01-06

**Authors:** Nathalie Alazard-Dany, Solène Denolly, Bertrand Boson, François-Loïc Cosset

**Affiliations:** CIRI—Centre International de Recherche en Infectiologie, Univ Lyon, Université Claude Bernard Lyon 1, Inserm, U1111, CNRS, UMR5308, ENS Lyon, F-69007 Lyon, France; nathalie.alazard-dany@inserm.fr (N.A.-D.); solene.denolly@ens-lyon.fr (S.D.); bertrand.boson@ens-lyon.fr (B.B.)

**Keywords:** HCV, DAA, antiviral targets

## Abstract

Hepatitis C infection is the leading cause of liver diseases worldwide and a major health concern that affects an estimated 3% of the global population. Novel therapies available since 2014 and 2017 are very efficient and the WHO considers HCV eradication possible by the year 2030. These treatments are based on the so-called direct acting antivirals (DAAs) that have been developed through research efforts by academia and industry since the 1990s. After a brief overview of the HCV life cycle, we describe here the functions of the different targets of current DAAs, the mode of action of these DAAs and potential future inhibitors.

## 1. Introduction

Hepatitis C Virus (HCV), the leading cause of chronic liver disease worldwide, is a positive-sense single-strand RNA virus classified in the *Flaviviridae* family in the *Hepacivirus* genus [1]. The fight against the disease started even before this agent was identified in 1989, with the use of interferon (IFN) since 1986 which was until recently the standard care of the treatment against HCV infection [2,3]. The therapeutical success of this treatment, aimed at stimulating host antiviral responses to eliminate the virus, was assessed by monitoring sustained virological responses (SVR), as defined by undetectable HCV RNA levels in the blood 12 or 24 weeks after the end of treatment. The IFN treatment was improved in 1998 with the addition of ribavirin, a non-specific antiviral agent, and in 2001, by adding polyethylene glycol to interferon molecules (PEG-IFN) [4,5,6,7]. The main problem with IFN-based therapies is that SVR rates remain rather modest, especially for the most common HCV genotype worldwide, and are accompanied by considerable adverse effects, making long treatment duration hard to support.

In the 2010s, the health authorities approved a succession of new medicines called direct-acting antivirals (DAAs). These molecules opened a new era in the treatment of HCV, achieving higher rates of SVR for most viral genotypes, with shorter treatment durations and fewer side effects. As their name suggests, DAAs directly target viral proteins that are essential for virus replication. After an outlook of the mains steps of the HCV life cycle, we will review the main targets of the marketed DAAs and those currently under development. The results of clinical trials are not addressed here, but are reviewed elsewhere [8].

The two main challenges when using DAAs, as experienced in the fight against HIV, are to treat all genotypes and to fight the appearance of resistance. It is particularly true for HCV, for which genetic variability is illustrated by the existence of seven genotypes and more than 80 different confirmed subtypes worldwide [1]. These genotypes and subtypes show different geographical distribution, pathogenesis and response to treatments. Whereas the first DAAs were directed against a single genotype, the new generation of DAAs target a greater variety of genotypes. Pangenotypic DAAs will be particularly interesting in low and middle-income countries as they will allow treatment of HCV patients without prior genotype testing. Extension of targets outside the hepacivirus is also envisioned by some researchers trying to develop antivirals active against different *Flaviviridae* [9].

HCVs high genetic variability is also a problem at the level of individuals. Because of the high replication rate and the lack of proofreading activity of the HCV RNA-dependent RNA-polymerase (vRdRp), HCV exists within its host as a population of slightly different viral variants, forming the “quasispecies” [10]. Some of the mutations induce amino acid changes that reduce the susceptibility to one or more antiviral drugs and are therefore called resistance-associated substitutions (RASs). Viruses harboring one or more RAS are called resistance-associated variants (RAVs) and are frequently associated with DAAs treatment failure if their fitness is sufficient [11]. RAVs can develop during treatment or may pre-exist as naturally occurring variants, albeit at low but sometimes clinically relevant levels, as reviewed in [12]. In both cases, RAVs selected during treatment and pre-existing RAVs contribute to the failure of treatments. The number of mutations necessary for a virus to become resistant and the probability that these mutations are selected in the presence of the drug is called the genetic barrier [13]. In addition to being pangenotypic, new antivirals are therefore developed with the aim of having high genetic barriers to resistance. The use of a combination of antivirals with different targets, each of them with high potency and high genetic barrier, now allows a high success of IFN-free oral regimens HCV treatment.

## 2. Overview of the HCV Life Cycle

### 2.1. Entry of HCV Particle into Hepatocytes

**Figure 1:**
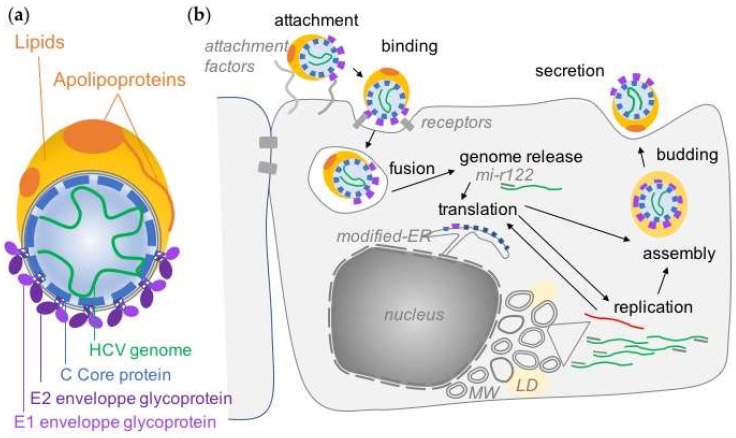
HCV lipoviroparticles and the virus life cycle. (**a**) HCV particles contain a positive strand RNA genome (in green) associated with Core proteins, enveloped by a membrane in which E1 and E2 glycoproteins are embedded and are tightly associated with lipids and apolipoproteins. (**b**) HCV life cycle. The different steps of HCV life cycle are indicated in black. ER Endoplasmic Reticulum. MW Membranous Web. LD Lipid Droplets. The negative strand replication intermediate in red.

HCV particles are 50–80 nm in diameter and have the particularity of being associated with neutral lipids (cholesterol ester and triglycerides) and apolipoproteins, which confers them their unusually low buoyant density (Figure 1a) [14,15]. HCV particles contain a positive single-strand RNA genome in close association with the core proteins, enveloped by a lipid membrane in which the two viral glycoproteins E1 and E2 are anchored. Association of particles with lipids tends to mask the viral glycoproteins but are thought to play a role in virus entry [16], at least in the initial phase of cell attachment, during which the interactions of apolipoprotein E with cell surface heparan sulfate proteoglycans [16,17,18] or with SR-BI have been implicated [19] (Figure 1b).

HCV E1 and E2 envelope proteins play a major role in virus entry and therefore in virus tropism to hepatocytes. Overcoming the initial difficulty to obtain HCV viral particles, HCV entry was first characterized with the use of HCV pseudo-particles (HCVpp) harboring E1 and E2 glycoproteins [20,21]. E1 and E2 act as complexes of disulfide-bound heterodimers and E2 is clearly identified to be responsible for receptor binding [22,23], whereas E1 seems to be an important component of an atypical fusion machinery, as reviewed recently in [24]. HCV entry is a complex multistep process in which different cellular proteins have been demonstrated to be involved. A cascade of interactions/co-interactions with the Scavenger Receptor class B type I (SR-BI) [23], the human Cluster of Differentiation 81 (CD81) [22] and the tight junction proteins claudin-1 (CLDN1) [25] and Occludin (OCLN) [26] has been described. These factors constitute the minimal set of essential entry receptors that prime the E1-E2 complex for virus entry [27]. This process is regulated by multiple host signaling pathways, in which, for example, epidermal growth factor receptor (EGF-R) is implicated [28]. Additional accessory entry co-factors and/or regulators are constantly described, as recently reviewed in [29].

Following attachment, HCV particles are internalized via the endocytic pathway and membrane fusion is dependent on endosomal acidification (Figure 1b) [30]. In the organism, cell-to-cell spread could be an important mechanism that seems to involve similar actors [31,32].

### 2.2. HCV RNA Translation and Replication

**Figure 2:**
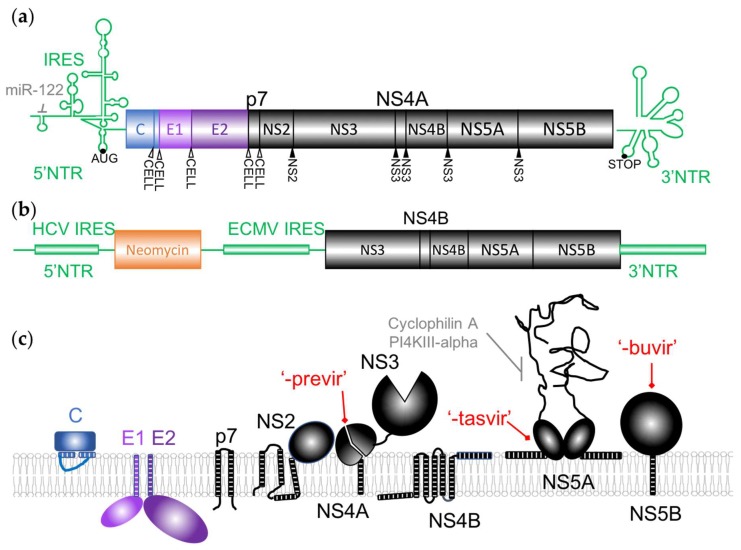
HCV virus and replicon organization and membrane organization of the viral proteins. (**a**) The HCV genome is a positive strand RNA containing a single open-reading frame (from AUG to stop) surrounded by 5′ and 3′ highly structured non-translated regions (NTRs). Translation of the polyprotein is enhanced by miR-122 binding in the 5′NTR as indicated. Translation is initiated at the internal ribosomal entry site (IRES). The polyprotein is cleaved by cellular (white arrowheads) or viral (black arrowhead) proteases. C: Core protein. All Non-Structural (NS) proteins (including p7) are in black. (**b**) Example of organization of a HCV subgenomic replicon that were extensively used for antiviral drug screenings, they contain all the proteins necessary and sufficient for HCV RNA replication. Neomycin: Neomycin resistance gene. ECMV: Encephalomyocarditis Virus. (**c**) Membrane association of HCV proteins. Hatched bars represent amphipathic alpha helix or transmembrane domains anchoring the proteins to the membrane. Two examples of essential cellular proteins interacting with NS5A are shown. The three main types of inhibitors used in the clinic (indicated in red by their suffix) are indicated next to their target.

The incoming viral RNA, approximately 9.6 kb in length, contains a single open-reading frame flanked by highly structured non-translated regions (NTRs) (Figure 2a) [2]. It is translated as a single polyprotein that is co- and post-translationally processed by both viral and host proteases, as indicated in Figure 2a, raising ten proteins. The Core, E1 and E2 structural proteins, present in the virions, are produced from the N-terminal end whereas seven nonstructural (NS) proteins are expressed from the C-terminus of the polyprotein.

The NTRs contain the main cis-acting elements that are essential for genome translation and replication, including an internal ribosomal entry site (IRES) in the 5′ NTR (Figure 2a) [33,34]. An abundant liver specific miRNA, miR-122, interacting with these sequences was demonstrated to regulate HCV RNA levels in cell culture, stimulating translation and genome replication [35,36]. This microRNA acts in an unconventional way by recruiting Argonaute 2 to the 5′ end of the viral genome, stabilizing and protecting it from degradation by 5′ exonucleases [37]. As it is essential for HCV replication, it was identified as a potential antiviral target. The viral RNA is also the template for viral replication that produces positive genomes used for additional protein production and viral assembly through a negative-sense intermediate (Figure 1b). NS5B is the viral RNA-dependent RNA polymerase (vRdRp), the key enzyme of RNA synthesis. When subgenomic replicons were first established (Figure 2b), the minimal translation and replication machinery was demonstrated to require the NTRs and NS3/4A, NS4B, NS5A in addition to NS5B [38]. As described below, these replicons were invaluable tools for early anti-HCV drug screening and discovery.

The function of the viral proteins targeted by DAAs will be briefly described here and in more details in the following sections. NS2 is a viral protease that contributes to the viral polyprotein maturation as well as to the assembly of viral particles. NS3 is both a viral protease involved in proteolysis of the downstream part of the polyprotein and a helicase. NS4A is a cofactor interacting with NS3 and anchoring it to the membrane. p7 was classified as a viroporin and has multiple roles in the assembly and secretion of virions [39]. As illustrated in Figure 2c, most of the viral proteins are membrane-associated and have several functions linked to the reorganization of the cellular membranes required for the replication of the HCV genome or later, during viral assembly. The massive rearrangement of cellular membranes after HCV infection consists of a “membranous web” mainly constituted early in infection by double-membrane vesicles (DMV) originating from the endoplasmic reticulum (ER) (Figure 1b) [40]. Most of the proteins of the replication complex are necessary for this process, but NS4B and NS5A are the major actors in the “membranous web” biogenesis and maintenance that contain the replication factories of the virus [41]. Several cellular factors are also recruited by the viral proteins to allow this process.

Describing the huge number of host factors that have been implicated in HCV replication is beyond the scope of this review but can be found elsewhere [42]. However, we will mention a few targets of particular importance in the context of antiviral research. Cyclophilins, cellular peptidyl-propyl cis-trans isomerase, were, for example, described as important co-factors for HCV and other viruses replication, as shown using cyclophilin inhibitors developed against HIV [43]. Cyclophilin A interacts with NS5A and is essential for the “membranous web” formation [44,45]. Phosphatidyinositol-4-phosphate-kinase-III alpha is another central host factor interacting with NS5A, inducing accumulation of PI4P within the membranous web [46], and provides an example of the complex imbrication between the HCV life cycle and lipid metabolism [17].

### 2.3. HCV Assembly, Budding and Secretion

Since 2005, the development of HCV molecular clones that recapitulate all the steps of the virus life cycle allowed much progress in the understanding of HCV assembly, budding and secretion [47,48,49]. Viral assembly is, however, still not fully described, as the models do not reflect all the interactions with liver lipid metabolism and generate particles that poorly resemble those produced in infected individuals [16,47,50].

Viral assembly occurs near replication complexes at assembly sites associated to ER-derived membranes, in close proximity to lipid droplets where core proteins can accumulate (Figure 1b) [51,52]. NS5A has been identified as a key player in the transition between viral replication and assembly, in addition to its role in replication, and was demonstrated to be involved in RNA genome delivery to the Core proteins [53,54]. NS2 and p7 also play a central role in virus assembly through their different interactions with structural and non-structural proteins. Most notably, they coordinate the recruitment of Core and the envelope glycoproteins E1E2 as well as NS3 and NS5A to the assembly sites, allowing the gathering of all viral actors for assembly correctly in the same place [55,56,57,58,59,60]. The small p7 viroporin was demonstrated to act in concert with NS2 in this process as well as being important at the later stage of viral envelopment [58,59,61]. NS4B was also demonstrated to play a role in HCV assembly [62]. Besides viral proteins, several cellular proteins have been described as main factors for HCV assembly, such as DGAT1 [63,64] and ESCRT proteins [65,66,67,68].

Viral particles acquire their envelope by budding at the ER. As mentioned above, lipid composition of the viral particles resembles that of VLDLs and HCV virion formation, and release may highjack their assembly pathway [69]. Maturation of the lipoviroparticles may also require an unconventional passage through the Golgi apparatus and a trans-endosomal secretory route (reviewed in [52]), but also occurs at a post-egress step [16,70]. p7, ApoE and numerous host proteins known to be involved in theses pathways are necessary for this process.

The short overview of HCV life cycle presented here provides a striking example of the fact that viral proteins are multi-purpose and exert several functions in the viral replication cycle, thus reflecting genetic economy. In an attempt to fight HCV infection, most of these proteins therefore stand as potential antiviral targets, as they are indispensable for one or more steps of the viral cycle. Before discussing the possibility to target viral entry or assembly, we will review the three main targets of DAA treatments, which are mainly involved in virus replication: NS3/4A, NS5B and NS5A.

## 3. Current Antiviral Targets

### 3.1. NS3/4A Inhibitors

The experience of antiprotease development in the HIV field facilitated the early development of drugs inhibiting NS3, as well as the fact that in vitro models were already available and complete replication systems were not necessary for preliminary screenings. The protease domain of the protein was targeted by the first HCV antivirals that were introduced on the market and by the currently recommended new DAAs. These molecules are suffixed -previr and are often collectively referred to as proteases inhibitors (PIs). NS3 protease activity plays a central role in the viral polyprotein maturation, as it cleaves four peptide junctions between the non-structural proteins, in complex with NS4A (Figure 2) [71,72,73]. The protease activity is also implicated in the modulation of the antiviral innate immune response [74]. The structure of the protease domain revealed a typical chymotrypsin-like fold, trypsin-like proteinase with two β-barrels and a highly conserved catalytic triad (His-57, Asp-81, Ser-139), and a binding site for the peptidic NS4A cofactor that is distant from the catalytic site [75,76]. NS4A is an essential cofactor for NS3 enzymatic activities that helps to localize NS3 to the membrane and anchors the HCV replication complex at the cellular membrane [77,78,79].

In addition to its typical serine protease domain, NS3 contains a short N-terminal amphipathic α helix (designated Helix α_0_) and a C-terminal helicase/NTPase domain, which is implicated in several critical steps of the virus life cycle [75,80]. Helix α_0_ plays an important role in membrane association, subcellular localization and stability of the NS3/NS4A complex, and participates in HCV virion morphogenesis [77,81,82]. The helicase/NTPAse domain was demonstrated to bind and unwind RNA duplexes during viral genome replication, making NS3/4A complex an important component of the viral replication complex, also implicated in virus particle assembly [78,82,83]. Drugs targeting functions other than the protease, for example helicase activity, have already been envisioned and could be developed in the future [84]. As NS3 and NS4A, but also the different NS3 domains, do not act independently, the inhibitors can affect several functions of the complex, allowing inhibition of multiple steps by targeting a single molecule [85,86,87].

Pioneer works on inhibition of NS3/4a protease activity identified cleavage substrate products as potential PIs [88,89]. Boceprevir and Telaprevir, the first two DAAs that benefited from a marketing approval in 2011, were both peptidomimetic linear ketoamides that bind the active site of the protease domain of NS3 [90,91]. Both DAAs were used in combination with PEG-IFN and ribavirin, and were soon discontinued in most countries due to their low genetic barriers to resistance, restriction to genotype 1 virus and severe adverse side effects [92]. Further development of PIs resulted in improved antiviral potency and approval of the main PIs recommended to date in different countries, the PIs being: Glecaprevir, Paritaprevir, Grazoprevir and Voxilaprevir [93,94]. The available crystal structures of NS3 in complex with candidate compounds facilitated structure-based design that has been extensively used to confront the issues of potency, resistance and pharmacokinetics, leading to the development of many new molecules, as reviewed in [8]. In terms of resistance, preferential loci of RASs were identified with the first generation of PIs and were taken into account for the optimization of the next DAA generation [95,96]. The efficiency of these molecules, especially in combination with DAAs targeting other viral proteins, allowed the development and approval of long awaited IFN-free regimen treatments with particularly high SVR and low side effects [97].

### 3.2. NS5B Inhibitors

The function of the NS5B protein at the 3′-end of the genome was evident as soon as the sequence of the HCV genome became available, on the basis of sequence comparison with other viruses [98]. The biochemical activity of the NS5B protein as a replicase was confirmed soon after [99] as well as the secondary structure of its catalytic site, which was found to be typical of a vRdRp: a right-hand like structure, with fingers, palm and thumb subdomains [100,101,102]. The NS5B protein, however, shows some particularities: a beta-hairpin loop insertion in the thumb domain thought to modulate the polymerase activity and an hydrophobic C-terminal domain, anchoring the protein to membranes [103]. The vRdRp was efficiently produced in different systems without this anchor, allowing further characterization of the protein properties [104,105]. Playing such an important role in the viral life cycle and given the wealth of information available for related proteins, the NS5B polymerase was identified very early as a target of choice for antiviral development and is certainly one of the best characterized HCV enzyme, as reviewed in [106]. Two types of inhibitors that target the catalytic or non-catalytic sites of this protein have been developed.

Based on knowledge about cellular and viral polymerases, the first inhibitors developed against NS5B were nucleo(s)tides inhibitors (NIs). These inhibitors mimic the natural substrate of the enzyme—they are typically incorporated into the nascent RNA chains and terminate RNA synthesis. Their development is, however, complicated by their instability and tendency to be inactivated by the cellular metabolism. Sofosbuvir is an uridine analogue, the first NI to be approved by the FDA in 2013, and still part of most of the recommended treatment combinations [107]. The real challenge during the development of Sofosbuvir was to understand the metabolism of the candidate inhibitors by liver enzymes to find the optimal formulation for a prodrug [108]. Short treatment durations of HCV treatments should prevent the side-effects, due to the known tendency of NIs to have cumulative toxicity [109]. A major concern during antiviral drug development is the appearance of resistance, particularly in viruses like HCV, whose vRdRp was demonstrated to be error-prone. The catalytic site of the vRdRp is, however, highly conserved between genotypes, suggesting the pangenotypic potential of such a drug and a minimal risk of the emergence of viral resistance. These hypotheses were confirmed by the analysis of clinical data demonstrating the extremely rare appearance of resistance mutations against NIs, which usually induce an important reduction in viral fitness [110,111]. With its favorable safety and tolerability profile, its high genetic barrier to resistance and its activity against most genotypes, Sofosbuvir is an antiviral of choice in most current HCV treatment combinations.

The second class of inhibitors, referred to as non-nucleoside inhibitors (NNIs), do not bind to the catalytic site of the enzyme [106]. They were discovered as inhibitors of the polymerase activity in screens using the replicon system, when resistance mutations were discovered in NS5B outside the catalytic site. These inhibitors exert their effect by inhibiting conformational changes required for polymerase activity. With the drugs tested to date, five different binding sites have been discovered—two in the palm, two in the thumb and one in the beta-hairpin chain of NS5B. Dasabuvir, the only NNI approved to date by the FDA, targets the palm of NS5B and is recommended for genotype 1 treatment only [112]. Next generation NNIs are currently being tested and characterized. The mode of action of Tegobuvir, a promising member of the imidazopyridine class identified in phenotypic screens, was, for example, shown to covalently bind the NS5B beta-hairpin after activation by cellular enzymes [113]. NNIs target poorly conserved sequences and therefore show lower barrier to resistance than NIs and poor pangenotypic activity, even if these defaults are attenuated in the upcoming next generation NNIs. They are, however, already useful when used in combination with other more potent drugs in some clinical presentations, to reinforce the efficacy of the treatment and contribute to reduce treatment duration.

### 3.3. NS5A Inhibitors

NS5A has long been considered as a potential antiviral target, since it was known early to be essential for viral RNA replication [114] and later also for viral assembly. The protein comprises three domains separated by short low-complexity sequences [115]. The N-terminal domain I is the best organized and characterized. It was demonstrated to bind Zinc, to be well-structured, with a short amphipathic helix in the very N terminus necessary for membrane targeting and to be essential for genome replication [76,114,115]. The two C-terminal domains are, in contrast, predicted to be intrinsically disordered. NS5A is known to exist in differently phosphorylated forms and to interact with a huge number of viral and cellular proteins, including itself to form dimers and maybe oligomers.

In contrast to the above-mentioned inhibitors, NS5A inhibitors could not be developed using biochemical assays, as the protein has no known intrinsic enzymatic activity. Interesting molecules were identified during the screening of libraries of compounds by different companies. Daclatasvir (DCV) was, for example, identified by screening over a million of compounds against HCV replicons, eliminating from the sorting both the compounds active against other viruses—as they would not be specific enough—and those inhibiting the enzymatic activity of NS3 or NS5B [116]. The development of resistance to the best candidate was demonstrated to be associated with specific mutations in the domain I of NS5A, thereby identifying NS5A as the target of the inhibitor. In a preliminary clinical trial, a single dose of DCV resulted in a rapid virological response, demonstrating that NS5A was a promising target for anti-HCV therapy [116]. DCV was shown to bind NS5A close to the N-terminus of domain 1, at the junction with the amphipathic helix that anchors NS5A at the membranes, without perturbing the dimerization nor the stability of the protein [117,118,119,120]. It was further shown that DCV and DCV-related molecules inhibit the formation of new DMVs that contains the replication complexes [45,118]. Other compounds such as Ledipasvir and Ombitasvir have similar properties and the same binding site to NS5A [121,122]. Second generation inhibitors were tested and/or optimized to be pangenotypic, active against previously identified NS5A RAVs and common NS5A polymorphs, and have reduced toxicity, as exemplified by Elbasvir and Velpatasvir [123,124].

Better understanding of NS5A functions is now coming from studies using the inhibitors, as reviewed in [125,126]. As screening of the compounds has been made on replicons, the studies initially focused on their impact on virus replication and cellular partners. As mentioned earlier, NS5A was shown to play an important role in the membranous web formation necessary for viral replication [41]. Tests on cell-infectious clones demonstrated that NS5A has multiple complementary activities in the virus life cycle, such as viral assembly [127] and more particularly on the transfer of the genome to the assembly sites [54]. Direct binding of NS5A inhibitors to NS5A has been reported, and a RAS was showed to act by decreasing the binding of DCV [120] and Ledipasvir [121] to NS5A. DCV was first described as inhibiting HCV replication by probably inhibiting the formation of double-membrane vesicles required for the replication of the viral genome [118]. In addition, some studies reported an action on assembly [127,128] by preventing the delivery of RNA to the core protein, the main component of the HCV nucleocapsid [54]. Description of the exact mechanisms of the inhibitors are still ongoing, but inhibition of NS5A seems to act on multiple steps of the viral life cycle, augmenting its effects.

## 4. Potential Future Targets

### 4.1. Targeting Other Viral Proteins

As mentioned in the introduction, all viral proteins essential for viral replication are considered as potential antiviral targets. The main strategy to combat resistance emergence is to combine inhibitors that target different proteins in order to limit the possibilities of appearance of cross-resistant mutations. This has led to research on alternative antiviral targets in addition to the above-described protease, polymerase and NS5A.

The small p7 non-structural protein is perhaps the most attractive of the potential alternative antiviral targets. In early studies using HCV replicons, p7 was found to be dispensable for viral replication [38]. But it soon became evident that p7 was essential for other steps of the virus-life cycle [129]. Studies of the functions of p7 became possible when the HCV cellular clones became available [130,131] and the protein was demonstrated to play a major role in the assembly and release of infectious HCV particles [44]. Indeed, p7 is a multifunctional protein that regulates the cellular secretory pathway as well as the interactions between viral proteins, and the localization of NS2 and core proteins towards capsid envelopment [55,56,58,59,60,61,132].

p7 is also able to form homo-oligomers that assemble to form ion channel structures and was therefore classified as a viroporin [44]. The precise role of this function, however, remains poorly defined but may be important for the regulation of the secretion of viral particles [131,133]. Viroporins are considered as an attractive class of antiviral targets, with an increasing number of such proteins described and characterized, and the success of using host ion channel inhibitors in other medical domains [134]. In addition, the same inhibitors can act on viroporins of different viruses and may lead to the finding of a broad-spectrum antiviral. The M2 viroporin of influenza A virus is the best studied example of this class of molecule [135]. M2 was also the target of the adamantane class of inhibitors that have been extensively used against Influenza A infection since the 1960s, even though their current use is now limited due to the appearance of widespread resistance. Although p7 is quite different from M2, it was shown to be inhibited by this same class of inhibitors [136], even though clinical trials in combination with IFN treatment showed no beneficial effects [137]. Other p7 inhibitors have been described or designed like, for example, GSK-2, hexamethylamiloride (HMA) or BIT225 [138,139,140,141]. BIT225 was of particular interest since it is able to block both p7 and Vpu from HIV, which might be a way to treat HCV/HIV co-infected patients. All these inhibitors have different efficiencies depending on the genotype of HCV [136] and a pangenotypic p7 inhibitor is still not available.

The last two nonstructural proteins, NS2 and NS4B, have also been targeted in preliminary studies [142,143,144]. Despite their potential utility and complementarity to the current treatments, these molecules may be not developed further for the moment as the currently existing treatments are judged by many as sufficient.

### 4.2. Host-Targeting Agents against Viral Replication and Entry

The appearance of resistance to antivirals in HCV, a highly mutable genome, could limit the future success of all the above-mentioned DAAs. Increasing the number of viral targets to fight the appearance of resistances is the main option to date, but targeting of some host factors that have been described as crucial for viral replication or entry has long been envisioned. The main advantages of these so-called host-targeting antivirals (HTA) over DAAs are a higher genetic barrier to resistance and their pangenotypic antiviral activity. The improved knowledge of virus–host interaction has led to the identification of potential targets such as cyclophilin and miR-122 inhibitors [145,146].

Interferon, the first historical treatment against HCV, was in fact a member of a special class of HTA sometimes referred to as immunomodulating proteins. Cyclophilin A inhibitors, derived or not from cyclosporins, immunosuppressive molecules that were isolated from fungus, were demonstrated early to have potent anti-HCV activities [43]. Once devoid of their immunosuppressive properties, these inhibitors were the first HTAs to go to the clinic and were demonstrated to have some potential. Cyclophilins are a group of multifunctional cellular proteins involved in protein folding, trafficking and cell signaling. The mode of action of cyclophilin A inhibitors is to disrupt its interaction with NS5A and prevent MVB biogenesis [44,45]. Because some Cyclophilin A inhibitors were also demonstrated to restrict HIV replication, they could potentially be of interest to treat patients with HIV/HCV co-infection [147]. Host proteins are not the only possible targets—clinical proof-of-concept studies have demonstrated that miR-122 inhibitors efficiently reduce viral load in chronically infected HCV patients [148,149]. However, these inhibitors, which require parenteral administration, are unlikely to be developed further in the context of efficient oral regimen. Another problem could be that targeting such an important molecule in liver function may have some impact on liver physiology and deleterious side effects.

HTAs are less specific than highly efficient DAAs that were faster into the clinic, but this is also one of their advantages. In addition to their pangenotypic potential in the HCV field, they could also lead to the discovery of broad-spectrum antivirals, since the host targeted protein can be implicated in the replication of other viruses. Targeting host lipid synthesis and metabolism to inhibit viral replication (and limit liver disease progression in the case of HCV) is, for example, envisioned against both HCV and Dengue virus [150]. Some of these drugs emerge from repurposing strategies and therefore also have the advantage over DAAs to be obtained at low cost. One important limitation, however, is the risk that they alter important functions, and their side effects need therefore to be carefully addressed, even if short treatment duration in the HCV field could limit these effects.

No matter how effective molecules inhibiting viral replication by targeting viral or host factors are, they do not protect non-infected hepatocytes from infection. Preventing virus entry is, however, an important goal, especially in the context of graft reinfection after liver transplant, but also in preventive treatments or to fight persistent infection [151]. The understanding of the complex mechanisms of HCV entry in cells has increased a lot in the past few years, with the description of a number of receptor or co-factors which makes all of them potentially suitable targets [152]. The use of neutralizing antibodies against viral or host proteins demonstrated the feasibility to fight HCV in chronically infected animal models by targeting virus entry [153,154]. ITX5061, an antagonist of SR-BI, was even tested in a phase I trial to prevent liver graft reinfection, with encouraging effects [155]. Given the numerous essential cellular partners identified to date, all the entry steps (attachment, binding, fusion) could in principle be targeted by antivirals targeting virus entry [151].

## 5. Concluding Remarks

Considerable research efforts on HCV biology by academics and industrials has led to the discovery of highly efficient DAAs less than 25 years after the identification of the virus. This great achievement may lead to a progressive loss of interest in the development of further research in the field. Many potentially interesting molecules have already been halted at early stages of development and will certainly not be tested further. Sustained efforts are needed, however, if we are to reach the 2030 eradication objective set by the WHO.

In an eradication strategy context, DAAs cannot be the only arm of the plan. They are still high cost and therefore poorly available in low income countries where HCV incidence is high. This is not their only limitation. Treatment with DAAs may lead to the emergence of resistant strains, does not protect from reinfection and, as infection is asymptomatic, people unaware of their status continue to transmit the virus. In addition, the high efficiency rates still leave some patients without therapeutic options. These are now a minority of patients and often referred to as “hard-to-treat patients”; they are those who are co-infected with HBV, those who present liver or renal dysfunction and those who underwent liver transplantation or previous treatment failure [97,156]. Potentially deleterious drug–drug interactions with other treatments, against HIV for example, is also a major concern with the development and use of these new treatments [157]. Last but not least, the inhibitors on the market have not been used for a long time and the appearance of resistant strains is not unexpected, possibly leading to increasing the number or patients without treatment options in the future.

History and modeling suggest that HCV eradication will necessitate a vaccine. Progress in the understanding of the immune response to HCV has led to different vaccinal strategies, including DNA, peptides or recombinant proteins, vector-based vaccines, and virus-like particles or dendritic cell-based vaccination strategies; these are at various levels of development (recently reviewed in [158]). The challenge is to develop a vaccine eliciting both a broad T-cell response and neutralizing antibodies against a highly variable virus hidden in lipoviroparticles. The objective seems achievable in the next few years, if research in this direction remains still supported against this now “curable” disease.
